# Composite 2D Material-Based Pervaporation Membranes for Liquid Separation: A Review

**DOI:** 10.3390/molecules29122829

**Published:** 2024-06-13

**Authors:** Roberto Castro-Muñoz

**Affiliations:** Department of Sanitary Engineering, Faculty of Civil and Environmental Engineering, Gdansk University of Technology, 11/12 Narutowicza St., 80-233 Gdansk, Poland; food.biotechnology88@gmail.com or roberto.castro-munoz@pg.edu.pl

**Keywords:** desalination, ethanol purification, membrane processes, nanomaterials

## Abstract

Today, chemistry and nanotechnology cover molecular separations in liquid and gas states by aiding in the design of new nano-sized materials. In this regard, the synthesis and application of two-dimensional (2D) nanomaterials are current fields of research in which structurally defined 2D materials are being used in membrane separation either in self-standing membranes or composites with polymer phases. For instance, pervaporation (PV), as a highly selective technology for liquid separation, benefits from using 2D materials to selectively transport water or other solvent molecules. Therefore, this review paper offers an interesting update in revising the ongoing progress of PV membranes using 2D materials in several applications, including solvent purification (the removal of water from organic systems), organics removal (the removal of organic molecules diluted in water systems), and desalination (selective water transport from seawater). In general, recent reports from the past 3 years have been discussed and analyzed. Attention has been devoted to the proposed strategies and fabrication of membranes for the inclusion of 2D materials into polymer phases. Finally, the future trends and current research gaps are declared for the scientists in the field.

## 1. Introduction

Today, membrane technologies, such as microfiltration, ultrafiltration, nanofiltration, reverse osmosis [1], membrane distillation [2], pervaporation, membrane bioreactors [3], membrane gas separation, and membrane contactors [4], among others, are broadly applied in several fields due to their versatility in separating several compounds in gas or liquid phases. In principle, a typical membrane process uses a permeable and selective physical layer (called a membrane) to separate specific compounds from the feed bulk solution. So far, membrane processes have been applied in various applications such as methane purification, CO_2_ separation, water treatment and disinfection, the purification of solvents, seawater desalination, and food production processes, among many others [5,6,7,8,9].

Among membrane technologies, pervaporation (PV) stands as one of the most selective techniques in separating solvent molecules. Fundamentally, PV uses dense selective membranes to break azeotropic mixtures (either organic–organic, water–organic, or organic–water mixtures) and thus achieve the separation of compounds. The transport of permeating compounds is based on their solubility and diffusion (diffusivity) across interfaces (i.e., membrane) [10]. Here, the chemical nature of the membrane material (hydrophilic or hydrophobic) also becomes an essential property in separating polar and other molecules depending on their polarity. For instance, a hydrophilic membrane displays higher affinity to the most polar molecules than to non-polar ones (or less polar ones), while a hydrophobic membrane exhibits higher affinity to non-polar molecules (or less polar) than the most polar molecules.

PV technology has been applied in solvent purification (ethanol, isopropanol, methyl tert-butyl ether, etc.) [11], seawater desalination [12], the treatment of industrial liquid mixtures [13], aroma recovery, the dealcoholization of beverages [14], and to assist chemical and biochemical reactions. To some extent, PV has mainly been explored using polymeric membranes; however, inorganic-based membranes (including zeolite, carbon-based membranes, etc.) have also been evaluated in such applications. Today, as a current trend in the field, several inorganic materials, such as zeolites, MOFs, COFs, silicas, and 2D materials, are being introduced in polymer matrices to offset their limitations such as poor thermal stability, mechanical stability, and separation performance. The latter materials (2D materials) are becoming attractive in this field due to their hydrophilic properties and facilitated permeance of water. In recent years, the research community has reviewed the ongoing progress of using 2D materials in membranes for different membrane processes, such as ultrafiltration, nanofiltration, forward osmosis, membrane gas separation, and for water, gas, and ion separation [15,16,17]; however, such reviews did not provide information on the ongoing progress of such novel materials in PV membranes. Therefore, in this study, we review the advances made (over the past 3 years) in utilizing 2D materials in composite membranes for PV applications, including seawater desalination and water purification, solvent purification, and the removal of organics from aqueous systems. Besides providing the most relevant results in the field, this paper analyzes the current strategies regarding membrane fabrication and their effect on the resulting membranes. After reviewing the literature, future perspectives in the field are given.

## 2. Properties of 2D Materials in Molecular Separations

**Figure 1:**
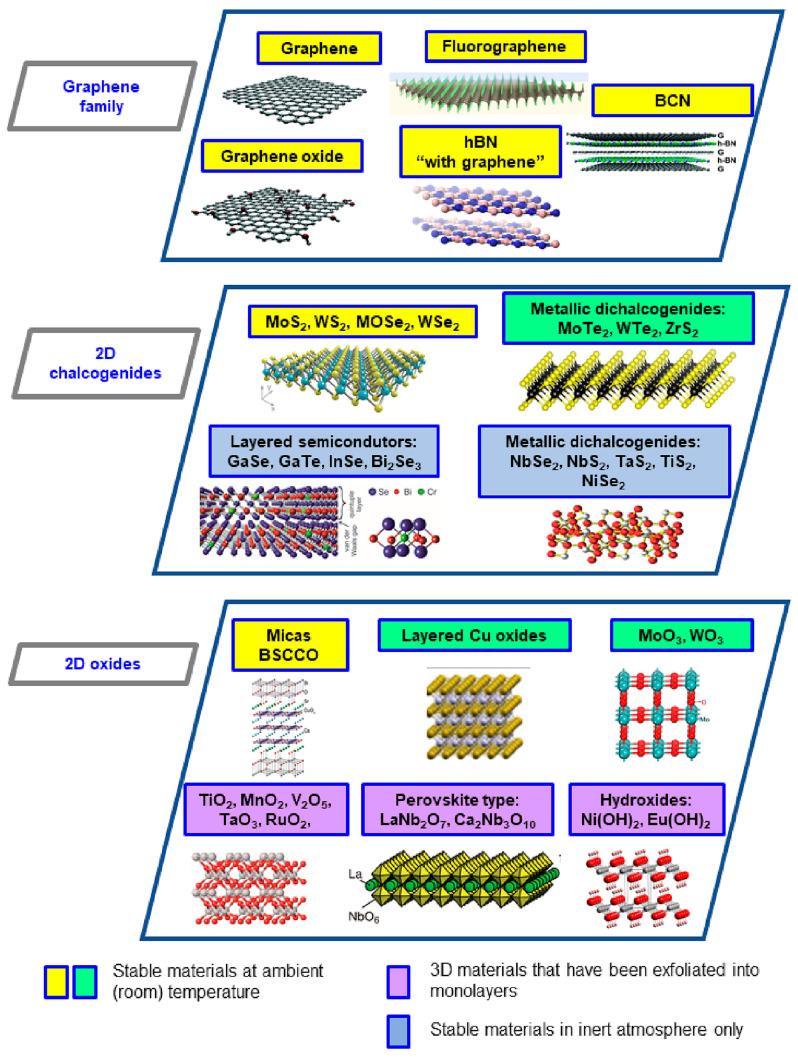
Classification and structure of 2D materials. Adapted from Castro-Muñoz [20].

Two-dimensional (2D) materials are revolutionizing science due to their applicability in various fields, such as biosensing, electronics, memory devices, insulators, and composite materials for processes, anticorrosion coatings, and energy storage [18,19], to mention just a few. These materials exhibit extraordinary features which make them suitable for promising applications depending on their structure. For instance, Figure 1 illustrates the classifications and provides some examples of developed 2D materials and their structures.

Unlike other inorganic materials (such as MOFs, COFs, and zeolites), some 2D materials (such as those in the graphene family) are attractive for water separation according to their intrinsic properties. For example, they present uniform-sized nanopores and apertures conferring an outperforming transport of water molecules when implemented in membranes and thus membrane processes [21]. Importantly, these materials can be precisely assembled in laminate form with a customized interlayer distance allowing for a superior molecular sieving effect to be carried out. Of course, such performance depends on the structural properties of the materials such as the pore size, d-spacing, and hydrophilicity. Interestingly, Table 1 covers the structural features of the most common 2D materials, along with their main separation mechanisms in liquid or vapor separations. 

Molecular sieving is the most associated separation mechanism in these materials. However, electrostatic interactions can also take place in molecular separations. To date, due to its hydrophilicity and facilitated water permeance, graphene oxide (GO) has been widely studied in water separation so far. When incorporated in polymer matrices, this material brings improved hydrophilicity in the resulting composite along with high permeation fluxes, while its outperforming separation relies on different interactions such as hydrogen bonding, electrostatic interactions, and π–π interactions [22,23,24]. On the other hand, hexagonal boron nitride (h-BN) can separate chemical species and pollutants thanks to different molecular interactions, such as electrostatic interactions, surface complexation, hydrophobic stacking interactions, chemical or physical adsorption, van der Waals forces, and hydrogen bonding [25]. Based on these findings, 2D materials represent an interesting option for preparing membranes in which water separation or purification is involved. The following section addresses the ongoing progress of utilizing 2D materials in specific PV applications, such as solvent purification, organics removal, and desalination. 

## 3. Ongoing Studies in 2D Materials for Composite PV Membrane Fabrication for Selective Molecular Separation

### 3.1. Solvent Purification

To date, PV has mainly been applied in the removal of component traces from solvent (solvent purification). As a clear example, PV is employed in the extraction of minimal water quantities from alcohols (ethanol and isopropanol) [26,27]. In this regard, if the aim is to separate water molecules from solvent matrices, the implementation of hydrophilic membranes is needed in which membranes preferentially transport water faster than polar organic membranes such as ethanol and isopropanol. Very recently, Castro-Muñoz et al. [28] reviewed the current advances in the purification of organics (mostly alcohols) using polymer PV membranes; however, the gap in the knowledge on the implementation of 2D materials in this application was not covered. To some extent, great advances have been documented over the past couple of years; for instance, Xu et al. [29] proposed two-dimensional MXene for the fabrication of permeable composite membranes with chitosan (CS), which were later tested for the dehydration of several organics (dimethyl carbonate, ethanol, and ethyl acetate). Interestingly, after the spin-coating of the MXene/CS dope solution, the membranes were dried and subjected to a crosslinking procedure for improved mechanical and chemical stability. The addition of 2D materials not only improved the physicochemical properties of the pristine CS membranes but also improved both permeability and selectivity in the PV experiments. For instance, in binary mixtures, the composite CS membranes filled with 3 wt.% MXene displayed total permeation between 1.4 and 1.5 kg∙m^−2^h^−1^ (at 50 °C) when separating water from ethanol, dimethyl carbonate, and ethyl acetate with separation values of 1421, 906, and 4898, respectively. According to the experimental findings, the authors noted that the addition of this 2D material did not improve the surface sorption, but its interlayer channels boosted water transport. After comparing the current concepts of membranes applied in this field, this study highlighted that these membranes are much better than others that contain other inorganic materials, such as Fe_3_O_4_, UiO-66, POSS, and MOF-801, as summarized in Table 2. However, it seems that GO intercalated membranes by UiO-66 exhibit much better performance than MXene/CS membranes [30].

The authors also concluded that, based on these findings, a laminar MXene membrane would presumably perform better in the removal of water from these organics via PV technology [29]. Of course, this assumption is valid if a laminar MXene membrane can be successfully fabricated. Somehow, MXene membranes stacked by MXene nanosheets were successfully prepared by Wu et al. [36]. Unfortunately, they did not perform better than Xu’ s membranes [29] for ethanol dehydration, reporting a selectivity value of 135 with a total permeation rate as low as 0.26 kg∙m^−2^h^−1^ (at 25 °C). It is worth mentioning that the authors experimented some degree of swelling degree in the MXene-based membrane when immersed in water/ethanol mixtures, which consequently enlarged the interlayer spacing. Also, membrane material spacing increased when immersed in pure water, but it displayed a much better anti-swelling effect in ethanol [36].

Very recently, Wang et al. [37] designed modified MXene–sodium alginate membranes with layered dihydroxide (LDH) for the effective extraction of water from ethanol. The purpose of this functionalization in GO was to substantially enlarge the d-spacing of GO and thus obtain an easy passage of water due to fast dissolution and diffusivity. When applied in PV, the hydrophilic composite membranes revealed a permeation of 1.3 kg∙m^−2^h^−1^, while selectivity was in the order of 1650. However, the authors pointed out that just the addition of 0.4 wt.% of the modified MXene into the polymeric membrane greatly improved the permeation by 80%, while the rejection of the modified MXene showed an improvement of 340%. Using double-layered LDH, Du and co-workers [38] vertically tailored an arrangement of LDH over polyacrylonitrile membrane support by means of an in situ hydrothermal protocol. This strategy resulted in intrinsic channels inside the membrane that were able to exclude water and ethanol. For water/ethanol separation, the composite membranes exhibited a selectivity value of 1903 and high-water permeability expressed in 7742 Barrer.

**Figure 2:**
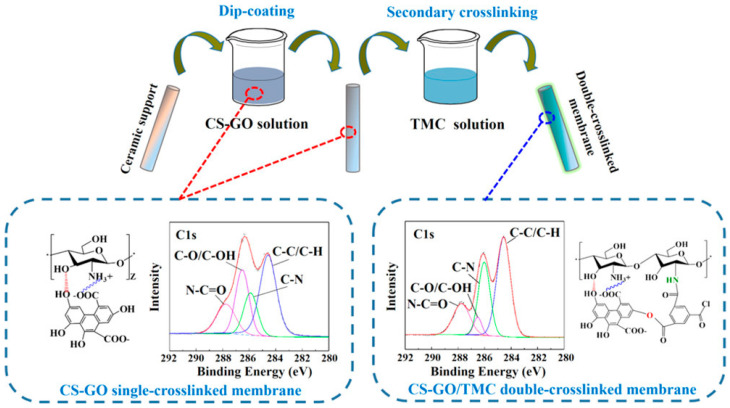
An illustration of the fabrication protocol of the GO-based membranes with double crosslinking and the interaction between GO, chitosan, and trimesoyl chloride. Adapted from [40].

Gallardo et al. [39] utilized another type of 2D material, like MoS_2_, as a filler for a thin-film composite membrane. However, the nanomaterial was preliminary functionalized with polydopamine. The resulting composite 2D membrane (thickness 0.07 µm) was tested in the removal of water from isopropanol using 30% water/isopropanol model mixture. The overall permeation was reported to be as high as 2.8 kg∙m^−2^h^−1^ (at room temperature), where the obtained permeate samples presented a water content of 99.14 wt.%. As for long-term operation, the composite membranes seemed to offer the same separation efficiency over 160 h of operation, but a worsened overall flux was noted with an initial reduction of 1.5 times in the first 20 h of operation. To some extent, this work opens a new window to explore the application of MoS_2_ material in composite membrane fabrication for PV separation. In comparison with Gallardo’s membranes [39], double crosslinked GO membranes with chitosan and trimesoyl chloride yielded better separation efficiency and permeation for isopropanol purification (10 wt.% water in alcohol) [40]. The fabrication procedure of such GO-based membranes is demonstrated in Figure 2.

In these membranes, the interaction between polar sites (hydroxyl) of chitosan occurred while similar polar sites were reacted with acryl chloride groups with trimesoyl chloride (see Figure 2); simultaneous chemical boding resulted in extensive stable membranes for molecular separation. Based on the author’s experimentation, the crosslinked composite loaded with only 0.1 wt.% GO achieved a permeation of 4.3 kg∙m^−2^h^−1^ while efficiently obtaining a separation factor value of 1491 [40]. The generated composite GO-based membranes also performed stably in a 100 h long test with impressive unchanged separation and permeation. In this field, it could be interesting to analyze how such membranes behave when separating water from other organics (e.g., ethanol, dimethyl carbonate, or ethyl acetate).

It has been documented that molecular transport in GO membranes relies on vertical transport across the GO defects and slit-like pores between adjacent sheets, along with lateral transport in the interlayer galleries. Therefore, molecular transport in GO membranes exhibits hierarchical water transport. Considering such insights, Dong et al. [41] tailored the hierarchical channel structure, including the slits, defects, and interlayer galleries, of a GO membrane via co-assembling pristine and etched GO nanosheets. For the dehydration of butanol, such membranes achieved a superior water/butanol separation factor of 5705 with a permeate flux of 4.81 kg∙m^−2^h^−1^. The authors mentioned that such separation performance has been attributed to several aspects, such as the replacement of partial GO with etched GO augmenting the number of slits between adjacent nanosheets owing to the smaller lateral size, as well as the number of defects on the nanosheets. Also, the increased slits/defects conferred shorter pathways for molecular transport, reduced molecular diffusion resistance, and increased permeance. As for the separation efficiency, the improved interlayer packing and interaction achieved after introducing etched GO provided a more ordered nanosheet assembly and elevated the anti-swelling capacity, thus narrowing the interlayer spacing and boosting the size-exclusion effect of interlayer channels towards butanol molecules. In a different work, the self-assembly of sulfonated carbon quantum dots and GO nanosheets was carried out by Xiong et al. [42], who synthesized highly hydrophilic two-dimensional sub-nanometer confinement interlayer channels for fast water transport. According to the authors, the nanodots acted as water transport promoters and crosslinkers, which contributed to the exceptional performance achieved in the dehydration of butanol, ethylene glycol, and isopropanol. For instance, the membrane with a thickness of 150 nm revealed a total flux of 5.88 kg∙m^−2^h^−1^ and a separation factor of 4407.

### 3.2. Organics Removal from Water or Organic Mixtures

When dealing with the removal of diluted organics from aqueous solutions, it is necessary to use hydrophobic membranes, which preferentially allow for the transport of non-polar to less polar components compared to water. However, when dealing with the extraction of organic compounds contained in organic solutions, it is important to know the polarity of such organic compounds and thus select the ideal hydrophilic or hydrophobic membrane to remove the traces of the undesired organic components. For instance, GO was embedded in a hydrophilic polyimide to generate mixed matrix membranes, which were later evaluated for their ability to extract methanol (MeOH) diluted in methyl tert-butyl ether (MTBE). The latter compound is used as an additive in the production of lead-free gasoline; however, MTBE needs further purification as it contains residual MeOH from the reaction with isobutylene. Herein, the mixture forms an azeotrope with 14.3 wt.% of MeOH and 85.7 wt.% of MTBE. These traces of MEOH have been removed using GO/polyimide mixed matrix membranes [43]. Apart from increasing the flux in pristine polyimide membrane, GO was contributed to selectively remove MeOH, revealing a separation factor of 28. The total flux of the composite membrane filled with 4 wt.% of GO showed approximately 0.091 kg∙m^−2^h^−1^. 

**Figure 3:**
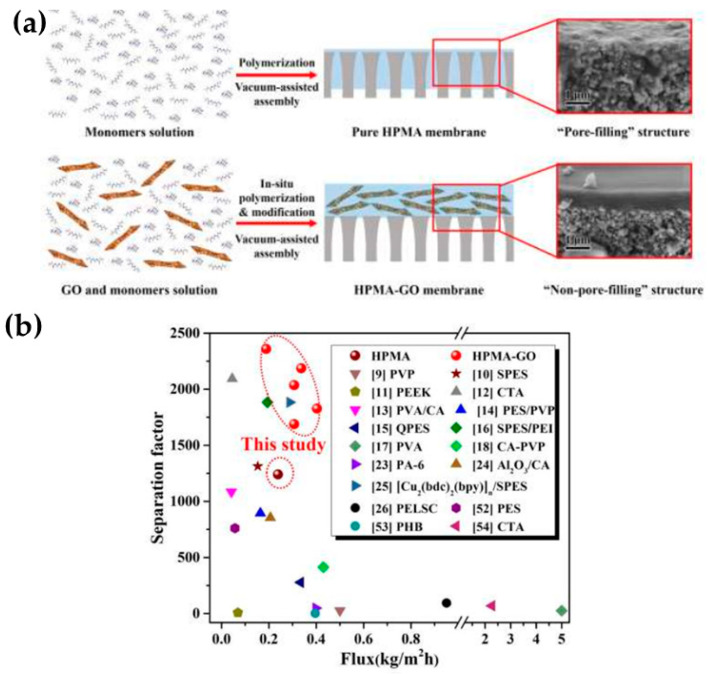
(**a**) A graphical depiction of the in-situ polymerization modification method for the dispersion and compatibility of GO in the polymer phase and (**b**) its comparison with the current state of the art in MeOH/MTBE separation via PV. Adapted from Wang et al. [44]. The red dots represent the findings from Wang’s study.

By separating MeOH from MTBE, Wang et al. [44] demonstrated better performance in GO/polymer membranes due to the production of MeOH transport channels with the embedding of GO into a polymerized hyperbranched polymer, as graphically illustrated in Figure 3a. Importantly, GO was chemically modified using two different alkylamines (octylamine and octadecylamine) and reduced in hydrazine monohydrate. Surprisingly, the resulting composite membranes had permeation between 0.2 and 0.4 kg∙m^−2^h^−1^ in the separation of a 10 wt.% MeOH/MTBE mixture (40 °C). This represents a greater value than the ones reported by Castro-Muñoz et al. [43]. Moreover, the separation efficiency was much better than that obtained in a later study, e.g., the separation factor values were between 1700 and 2400. It is worth mentioning that, apart from the improved separation performance and stability over 120 h of operation, the in-situ polymerization modification contributed to improve the dispersibility of GO nanosheets in the polymeric phase. As shown in Figure 3b, the GO/polymerized hyperbranched polymer composite membranes also demonstrated superior performance than most of the polymer membranes reported so far.

Towards the recovery of butanol diluted in water solutions, Alberto et al. [45] experimented with the hydrophobization of GO flakes with alkyl functionalization, which were later merged into PIM-1 polymer phase. Experimentally, the flux was improved by 40% thanks to the incorporation of functionalized GO nanosized materials, while the embedding of micrometer-sized ones resulted in worsened selectivity towards n-butanol when compared to pristine PIM-1 membranes. The latter finding suggests that the generated filler–polymer interface has a pivotal role in the overall separation performance in molecular separations like PV. For instance, when micrometer-sized materials are utilized, voids between the polymer and graphene take place, while smaller materials make their alignment easier with the polymer segments. This alignment potentially decreases the possibility of the appearance of non-selective and large voids. Apart from that, the PV performance of the membrane filled with small flakes tends to be compromised when loaded with higher than 0.1 wt.%, which can be credited to the effect of filler agglomeration. In general, the PV performance of the composite membranes was the best (separation factor of 17) when presenting 0.05 wt.% octyl-functionalized GO, while exhibiting an improvement of 40% in total permeation.

### 3.3. Seawater Desalination and Water Purification

Knowing the preferential transport of water into 2D materials, the latter materials have been extensively implemented into different membranes for removing salt ions and other species from water. Very recently, up-to-date information has been released in terms of the embedding of different 2D materials, including graphene and its oxidized version (GO), MXene, carbon, and boron nitrides, into polymer phases [20,46,47]. Similar to the extraction of water from organic solvents, GO has been one of the widely investigated materials in this application; for instance, when incorporated in hydrophilic chitosan membranes, it is able to provide a permeate flux as high as 30 kg∙m^−2^h^−1^ while displaying an almost complete rejection of salt (ca. 100%) (5 wt.% aqueous NaCl at 81 °C) [48]. Researchers have even noticed that the water/salt selectivity was improved by increasing the GO loading. Using a different polymer phase, exfoliated hydrophilic GO was embedded into PVA supported onto the PAN membrane support. According to the authors, the PVA polymeric chains acted as spacing molecular bridges to ensure the interaction of GO with the crosslinker [49]. The final membranes, exhibiting a thickness of 120 nm, reported a permeate flux higher than 69 kg∙m^−2^h^−1^, which represents more than double the thickness reported by Qian’s study [48]. The GO-filled PVA membranes were more permeable as they were much thinner while maintaining a similar rejection rate (99.9%) when operating at 70 °C (35 g L^−1^ NaCl) [49]. 

**Figure 4:**
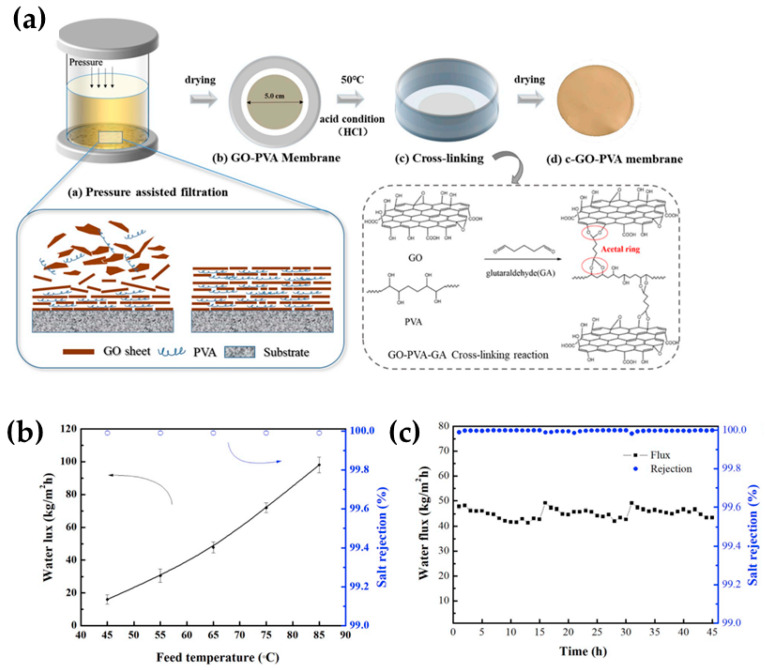
(**a**) A graphical illustration of the microstructured GO-PVA membranes and the crosslinker interactions with the materials. (**b**) The PV’s desalination performance depending on the operating temperature (**c**) and their long-term operation (10 wt.% NaCl salt aqueous model solution at 65 °C). Adapted from Sun et al. [51].

According to a recent review in seawater desalination via PV, PVA is the most suitable polymer in desalination due to its hydrophilic and water affinity properties, which are credited to its multiple hydroxyl groups; however, further stability must be given via crosslinker [50] or filler loading to restrict its polymer chains. By then, the highest desalination performance (water flux) ranged from 143 to 234 kg∙m^−2^h^−1^, where thin PVA membrane specimens were prepared; however, ultra-thin membranes doped with 2D materials, like MXenes and GO, also exhibited a high enough permeation between 85 and 98 kg∙m^−2^h^−1^. Among a similar range of water permeation, Sun et al. [51] engineered microstructure PVA-intercalated GO composites in seawater desalination, as illustrated in Figure 4a. The microstructured membranes exhibited a flux as high as 98 kg∙m^−2^h^−1^ and also yielded a salt rejection rate of approximately 99.99% for the desalination of a 10 wt.% NaCl aqueous model solution (at 85 °C) (see Figure 4b). To some extent, the permeation values these membranes exhibited depended on the temperature ranging from 45 to 85 °C, while the salt rejection values remained unchanged at ca. 100%. The authors reported that the permeable properties of the GO-PVA composite membranes depended on the 3D crosslinking network generated with GO sheets and the resultant interlayer spacing. As for long-term operation, such membranes showed a decrease from 48 to 43 kg∙m^−2^h^−1^ within the first 15 h while the rejection values were still unchanged, as shown in Figure 4c [51].

Compared with the literature, Sun’s membranes [51] overcome the performance of most GO based membranes in desalination using the PV process, as reported in Table 3. In general, GO membranes and polymer composites exhibit high salt rejection values (over 99%), while the water permeation flux varies depending on the polymer–GO interactions, which directly impact the final structural properties of the membranes. In any event, the membranes containing GO nanosheets exhibited attractive permeation rates. 

**Figure 5:**
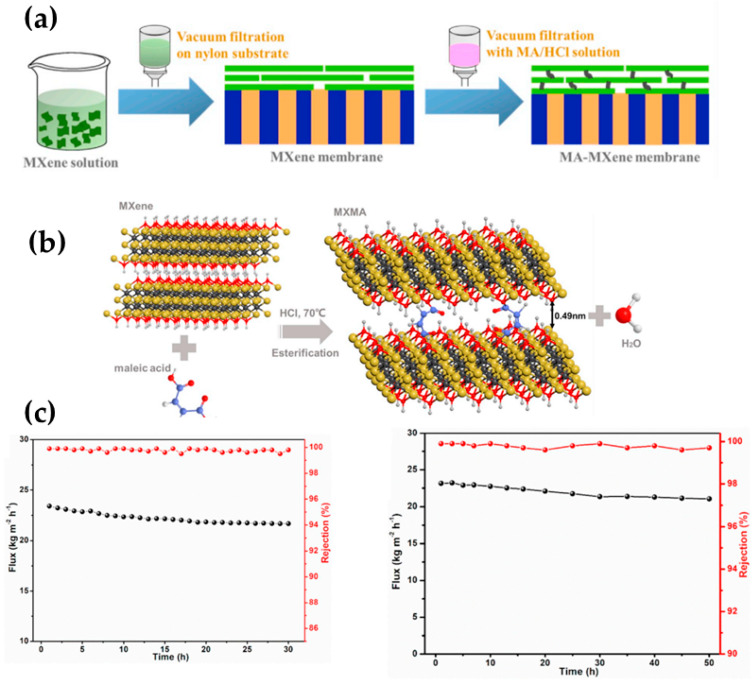
(**a**) A graphical depiction of the fabrication procedure of crosslinked MXene membranes using maleic acid, (**b**) the hypothetical interaction between MXene and maleic acid via esterification reaction, and (**c**) the long-term desalination pervaporation of maleic acid-crosslinked MXene 3.5 wt.% NaCl solution (30 °C, left graphic) and real seawater (30 °C, right graphic). Adapted from Ding et al. [47].

Considering that most 2D materials tend to be prone to d-spacing enlargement when immersed in water [55], researchers should be careful when performing long-term desalination performance as such materials may somehow present worsened performance. Considering the latter issue, Ding et al. [47] developed a 2D laminar maleic acid crosslinked composite MXene membrane, which showed tunable nano-sized channels, and they were later tested for seawater desalination by means of pervaporation. The fabrication procedure and mechanism of reaction between the 2D material and the crosslinker are depicted in Figure 5a and Figure 5b, respectively. As for the interaction of the organic acid and the inorganic 2D material, the polar hydroxyl (-OH) terminals on MXene interacted and reacted with the carboxyl (-COOH) terminals from the organic acid, generating ester bonds. This interaction contributed to a stable interlayer spacing of the 2D sheets and subsequently reinforced the material, thus restricting the structure of the resultant membrane toward the swelling in the aqueous system.

Importantly, the membranes maintained the intrinsic nanochannels, and a particular phenomenon was observed in such membranes; for instance, desalination with different thicknesses was performed (3.5 wt.% NaCl solution), and it was observed that the water transport (i.e., permeation) displayed a direct relationship with the membrane thickness. When the membrane thickness was decreased from 370 to 30 nm, water permeation proportionally resulted in an increase from 5.1 to 22.8 kg∙m^−2^h^−1^ thanks to the diminished mass transfer resistance, maintaining an ion rejection rate of over 99.7%. However, when the decrease in thickness continued when using smaller amounts of MXene and maleic acid solution, the resulting membrane revealed a scarce separation efficiency with an approximately 30% salt rejection rate. According to the membrane characterization, this has been related to the appearance of non-selective defects over the membrane surface. After this deep analysis, the authors concluded that a thickness of 30 nm resulted in a defect-free crosslinked MXene membrane. It is worth mentioning that the crosslinked membrane displayed a robust structure and integrity by offering long-term stability in pervaporation desalination in both the salt model solution and read seawater (see Figure 5c), while the non-crosslinked MXene membrane exhibited a worsened rejection and changeable water flux [47].

As part of the current state of the art of MXene membranes, Fareed et al. [56] synthesized thin (100 nm) MXene membranes, which were self-crosslinked due to dehydroxylation. Particularly, self-crosslinking consisted in the induction of Ti-O-Ti bonds thanks to the reverse hydrolysis of MXene. When tested for desalination in PV (100 g/L of salt solution), the membranes exhibiting an interlayer spacing of 1.43 nm yielded a water flux of 70 kg∙m^−2^h^−1^ at 70 °C. Importantly, these crosslinked MXene membrane specimens also offered a 99.9% salt rejection rate. Apart from the obtained PV desalination performance, these membranes benefited from self-crosslinking with a smaller swelling degree, allowing for the desalination process to be operated over 48 h with a permeation ranging from 49 to 57 kg∙m^−2^h^−1^ and with unchanged rejection rates. It is worth mentioning that these authors observed that these membranes exhibited a reduction in the interlayer spacing from the dry state to wet state with values ranging from 1.54 to 1.43 nm; the authors confirmed that such reduction was not attributed to momentaneous surface hydration, but it was credited to the production of the Ti-O-Ti structural bonding [56].

**Figure 6:**
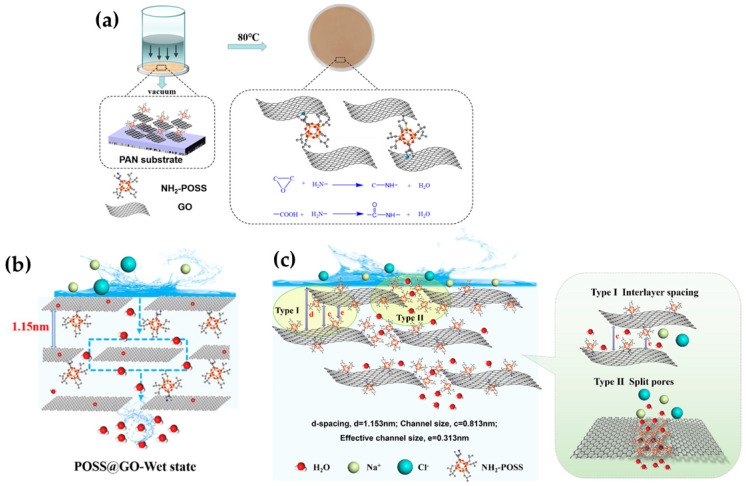
(**a**) A graphical depiction of the fabrication protocol of POSS@GO composite membranes and (**b**) their suggested mechanism of water transport, as well as (**c**) the ion separation mechanism. Adapted from Zhan et al. [57].

Zhan et al. [57] very recently modified the structure of GO to fabricate novel composite membranes for PV desalination. At this time, the authors strategically used aminopropylisobutyl modified polyhedral oligomeric silsesquioxane (NH_2_-POSS) to be intercalated inside the GO interlayers and subsequently applied glutaraldehyde, as a chemical crosslinker, to synthesize POSS@GO composite membranes. The synthesis protocol of the membranes is represented in Figure 6a. Likewise, the intercalation application of that intrinsic rigid nanocage (POSS) in the GO structure contributed to enlarging the d-spacing to favor the transport of water molecules to some extent while hindering ion rejection (ca. 99.98%). The high water flux of 112.7 kg∙m^−2^h^−1^ was obtained due to the facilitated mass transfer of water thanks to a capillary force generated in GO channels, as presented in Figure 6b. According to the authors, the composite-based GO exhibited a strong interaction with water due to its multiple oxygen units in oxidation regions. As for the rejection, the generated steric hindrance induced by the nanocage material could hinder the transport of the Na^+^ and Cl^−^ ions with a hydrated ion radius. As hypothesized in Figure 6c, the POSS material attached to the edges of GO could assemble and rearrange in the slit pores thanks to the flexible alkyl chains on the cages. However, merging the nanocage into a GO structure also contributed to reducing the swelling phenomenon in the resultant hybrid membrane; this enhanced resistance to swelling definitely contributed to obtaining a stable and outstanding desalination performance over 24 h at an elevated operating temperature (80 °C) [57]. In conclusion, this engineered nanoscale hybrid membrane demonstrates that the functionalization of 2D materials with hydrophobic properties may result in the enhancement of permeable properties while maintaining high rejection rates.

With similar salt rejection rates (99.98%) to those in Zhan’s study [57], Gurianov et al. [58] reported a rejection rate as high as 99.9% using modified GO membranes. In this work, the authors proposed the use of carbon-like spacers (such as fullerenols and nanoribbons) to modify the interlayer of GO. To some extent, these GO-modified membranes showed acceptable desalination performance, but their permeation rates were much lower (27 kg∙m^−2^h^−1^) than the previous development of Zhan’ membranes. 

It is worth mentioning that the proper oxidation of GO may result in high-performing GO membranes for desalination. According to Qian et al. [59], the oxidation degree may contribute to a desired interlayer structure for water transport. In their work, Qian and co-workers intensified the oxidation of GO by crosslinking it with borate in which crosslinking was governed by C–O–B bonds. Interestingly, the polar sites (hydroxyl) of GO were covalently linked with sodium borate, which resulted in the production of a highly selective and stable three-dimensional GO/borate structure (demonstrated with an over 100 h PV desalination test). The ultrathin membranes (138 nm) demonstrated complete salt rejection (99.9%) with a flux of over 91 kg∙m^−2^h^−1^ (10% salt solution at 85 °C). In a different work, bifunctional poly(dopamine–ethylenediamine) and a GO scaffold membrane were fabricated and chemically crosslinked by glutaraldehyde by Wang et al. [60], who proved that the GO backbone and hydrophilic functionalities allowed for unimpeded water transport across the novel membrane interface, which resulted in flux values of ca. 153 kg∙m^−2^h^−1^ with 99.9% salt rejection when desalinating salt solution with a 3.5 wt.% NaCl concentration. Apart from this application, the authors found that such membranes were able to display acceptable heavy metal adsorption toward uranium. Using polyethyleneimine as a crosslinker, Wang et al. [61] tuned the interlayer spacing of a lamellar GO membrane for the desalination of hypersaline brine. Impressively, the resultant crosslinked GO membrane showed a permeation flux of 86 kg∙m^−2^h^−1^ and a salt rejection rate of 99.99% in filtrating a 10 wt.% NaCl hypersaline solution (at 90 °C). The outstanding performance was credited to the polyethylen–imine intercalation, which broadened the interlayer spacing to decrease the transport resistance of the membrane while inducing the formation of more amide bonding and thus reducing the permeable void space of the membrane.

With the same idea of Qian et al. [59] toward the interlayer spacing regulation in GO via crosslinking, Jiao et al. [62] evaluated the effect of using a novel crosslinking agent, such as thiosemicarbazide, into GO membranes. The interaction of thiosemicarbazide and GO produced a covalent crosslinking reaction, allowing for the final composite membranes to exhibit a negative charge. At this point, the authors were able to control the spacing of the nanochannels of the 2D material; however, these membranes did not report an interesting permeation (approximately 7.8 kg∙m^−2^h^−1^) but met the standard salt rejection rate (99.9%) for this type of membranes. This high salt rejection rate was obtained due to the negative charges of the thiosemicarbazide crosslinked modified GO membranes, which were able to efficiently hinder negative ions. Herein, the authors also supposed the appearance of a possible Donnan effect. On the other hand, for the low flux rates, the membranes were relatively thicker (552 nm) than other GO-based membranes, limiting their permeation rate. Nevertheless, even if these thiosemicarbazide crosslinked modified GO membranes initially exhibited a low permeation (0.42 kg∙m^−2^h^−1^, at 30 °C), this was later intensified until a permeation of 7.8 kg m^−2^ h^−1^ was reached when operating the system at 70 °C.

## 4. Conclusions and Research Gaps

In this review, the relevant findings on using 2D materials for membrane fabrication for PV are elucidated. Mostly, 2D materials (such as GO and MXene) have been used in molecular separations. Due to their high affinity to water and unprecedented transport, such materials have mostly been used for the dehydration of organics and seawater desalination. When dealing with the removal of organic materials from water, the hydrophobization of 2D materials is required. Here, alkylamines (octylamine and octadecylamine) in GO functionalization have been used for extracting MeOH or butanol from aqueous solutions. 

Based on this paper, it can be confirmed that PVA is still the most preferred polymer phase in fabricating composite membranes. This is because it presents a suitable hydrophilicity and functional groups to interact with either crosslinkers or inorganic phases. Regarding the latter aspect, the use of GO in PV membranes for desalination has been deeply explored. Regarding the latter application, researchers are focused on regulating/controlling the thickness (smaller thickness for higher permeation rates) as well as the restriction of possible d-spacing enlargement of GO materials in water environments. In recent years, another emerging material, such as MXene, has also been explored in seawater desalination via PV in which the resulting membranes have exhibited excellent outcomes. Finally, more efforts should be made to research the effects of other 2D materials, such as hBN, MoS_2_, fluorographene, and carbon nitrides, among others (see Figure 1), in polymer phases or self-standing membranes for PV desalination. The use of composite membranes for the dehydration of isopropanol has recently been explored [39]. In any event, the latter materials have been deeply explored for use in other membrane technologies (forward osmosis, nanofiltration, vacuum filtration, and membrane distillation) and minimally in PV. As for seawater desalination, a current research gap involves adapting such 2D composite materials into more attractive membrane module configurations for possible large-scale processes, especially in desalination [63].

## Figures and Tables

**Table 1:** Structural features and separation mechanisms in most common 2D materials. Adapted from Castro-Muñoz [20].

2D Material	Estimated Pore Size	Interlayer Spacing	Nature	Separation Mechanism
MXene	19.5 nm	<2 Å	Hydrophilic	Size sieving effect
Fluorographene	0.2–0.9 nm	6.2 Å	Hydrophobic	Size sieving effect/ molecular interaction
Graphene	~0.2 nm	~1.42 Å	Hydrophobic	Size sieving effect
Graphene oxide	<0.3 nm	5–9 Å	Hydrophilic	Size sieving effect
MoS_2_	2.8 nm	0.615 nm	Hydrophobic	Electrostatic interactions
g-C_3_N_4_	~3 nm	3–6 Å	Hydrophilic	Size sieving effect/ electrostatic interactions
h-BN	~3.1 nm	3.34 Å	Hydrophobic	Size sieving effect

**Table 2:** Comparison of MXene/CS composite membranes with other composite membranes containing inorganic nanomaterials for removal of water from different organics.

Composite Membrane	Temperature (°C)	Water Concentration (wt.%)	Total Flux (kg∙m^−2^h^−1^)	Separation Factor	Reference
*Water/ethyl acetate mixtures*					
MXene/CS	50	2	1.471	4898	[29]
UiO-66@ graphene oxide	50	2	3.233	6951	[30]
Perfluorosulfonic acid-TEOS	40	2	0.205	496	[31]
*Water/ethanol mixtures*					
MXene/CS	50	10	1.424	1421	[29]
MOF-801/CS	70	10	1.937	2156	[30]
Graphene/CS	30	10	0.010	1093	[32]
Fe_3_O_4_/CS	75	10	1.024	1500	[33]
POSS/CS	60	10	0.270	30	[34]
*Water/dimethyl carbonate mixtures*					
MXene/CS	50	2	1.428	906	[29]
Graphene oxide	40	2	1.400	484	[35]

**Table 3:** A comparison of microstructured GO-PVA membranes with the current literature reporting other GO composite membranes for PV desalination.

Membrane Type:	Interlayer Spacing (nm)	NaCl Concentration (wt.%)	Operating Temperature (°C)	Permeation (kg∙m^−2^h^−1^)	Salt Rejection (%)	Ref.
GO-PVA	1.23	10	85	98	99.99	[51]
GO-PAN	-	3.5	90	65.1	99.8	[52]
CDA-GO composite	0.91	3.5	90	20.1	99.9	[53]
PDI-GOF	0.83	3.5	90	11.4	99.9	[54]
pPDA-GO composite	1.13	3.5	90	10.7	99.8	[53]
GO-PVA-GA-PAN	-	3.5	70	69.1	99.9	[49]

Acronyms: PAN (polyacrylonitrile), pPDA (*p*-polydopamine), GA (glutaraldehyde), PDI (1, 4-phenylene diisocyanate), GOF (graphene oxide framework), CDA (1, 4-cyclohyxanediamine).

## Data Availability

Not applicable.
